# How Cochlear Implant Rehabilitation Impacts the Therapeutic Strategy for Vestibular Schwannoma

**DOI:** 10.3390/audiolres13010012

**Published:** 2023-02-08

**Authors:** Francesco P. Iannacone, Francesca Visconti, Elisabetta Zanoletti

**Affiliations:** Department of Neuroscience DNS, Otolaryngology Section, University of Padova, 35100 Padova, Italy

**Keywords:** vestibular schwannoma, cochlear implant, hearing rehabilitation, neurofibromatosis

## Abstract

Background: Since both surgery and more conservative treatments show long-term outcomes in patients with VS, the current challenge in its therapeutic strategy is to offer a cure with lower functional morbidity in terms of facial and hearing preservation or the possibility of hearing rehabilitation with a cochlear implant. Methods: PubMed and Scopus databases were searched from 2017 to November 2022. Fifteen articles met our selection criteria: (1) patients with a diagnosis of VS, either sporadic or NF2-related; (2) simultaneous or sequential cochlear implantation; (3) specified audiological test results and follow-up timing. Conclusions: Although the level of evidence for the presently included studies is low due to either the study design or the lack of treatment consensus, CI rehabilitation is a promising option, especially in small VS with compromised hearing and as a salvage option after a failed attempt at hearing preservation surgery.

## 1. Introduction

Vestibular schwannoma (VS) is a benign tumor arising from the nerve sheath of the VIII cranial nerve. In the last decades, the global incidence has increased from the historical “1 per 100.000” to 3.0–5.2 per 100.000 person-years, with a lifetime prevalence of 1 in 100 persons [1]. The treatment involves surgery, with or without a hearing preservation attempt, radiotherapy (RT), and observation. Despite institutional preferences, which always affect the choice and represent a BIAS when comparing patient outcomes among different options, the issue of small VS is still the most debated.

Small VS, defined as a tumor less than or equal to 10 mm in size in the cerebellopontine angle (CPA) [2,3], is a well-tolerated disease, with varying degrees of hearing loss and variable growth. Today, early MRI diagnosis allows the diagnosis of a greater number of small tumors than in the past. Although non-surgical treatments in small VS are the most conservative therapy options [4], the current challenge is to offer a cure for the disease with the lowest rate of long-term morbidity, i.e., with no further functional losses beyond those that led to the diagnosis. Since long-term outcomes of observation and radiotherapy for facial and hearing preservation seemed to suffer the test of time [5,6], a current dilemma is emerging between early surgery as a way to promote the functional preservation of the facial nerve and hearing preservation or rehabilitation with a cochlear implant [7,8]. The concept that surgery on a small tumor promotes better rates of facial nerve preservation dates to the first decade of the new millennium [9], and this was evident in the following years [10,11]. In 2012, Kaltoft reported that in the whole course of observation, including failures that were operated on later at a bigger size than when diagnosed, worse facial nerve surgical outcomes were reported than in primary cases operated on at smaller sizes. The drawbacks of the observational strategy for facial nerve preservation were also supported by other authors [8,11] and led to an alternative option in the emerging role of early surgery [7,8]. In experienced centers, it offered better outcomes for small VS when compared with the long-term results of primary radiotherapy and far better outcomes when salvage surgery followed RT failures [12,13]. Hearing preservation is focused on the same perspective: excellent short–medium-term results with conservative therapies, but poor outcomes over the long term. Hearing rehabilitation with cochlear impant (CI) in VS-related hearing loss has increasingly achieved a place in the therapeutic strategy, but it became clear that it was feasible and, possibly, successful if proactively applied when the tumor was small.

The aim of this narrative review is to provide an up-to-date panoramic view of how the introduction of cochlear implantation has been affecting therapeutic strategies.

## 2. Materials and Methods

**Figure 1:**
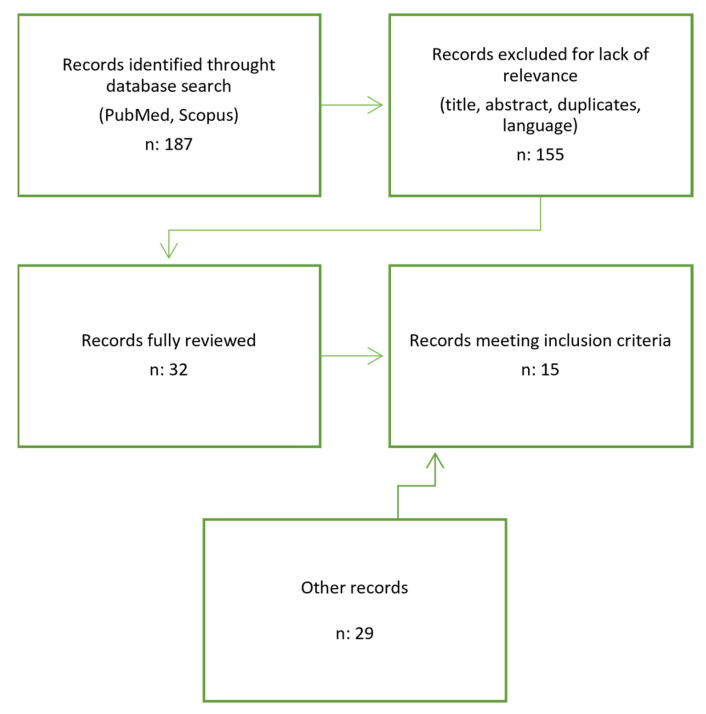
Diagram resembling Electronic Database Search and Inclusion/Exclusion process of the review. Year filter: January 2017 to November 2022.

This research was conducted using PubMed and Scopus. Combinations of sensitive and specific keywords, such as “Vestibular Schwannoma” and “Cochlear Implant”, were used. One hundred and eighty seven titles were reviewed independently from the authors, and thirty-two articles released in the last 5 years (from 2017 to November 2022) were selected and examined based on the title and abstract relevance; duplicates were removed, and only articles in the English language were retained. Out of 32 articles, 15 were included in the review because they fulfilled our selection criteria: (1) patients with a diagnosis of VS, either sporadic or NF2-related; (2) simultaneous or sequential cochlear implantation; (3) specified audiological test results and follow-up timing. Other articles were searched for in the references and databases when a particular aspect needed to be deepened. The chart in Figure 1 summarizes the article inclusion process in this review.

## 3. Results and Discussion

The main results of the studies are shown in Table 1.

### 3.1. Prerequisites in Management of VS and CI

Early surgery offers the best chance to preserve the cochlear nerve, and a crucial condition for cochlear implantation is the anatomic and functional integrity of the cochlear nerve. The macroscopic integrity of the VIII nerve is considered the decision-making factor after microsurgical tumor resection [15]. When preoperative hearing is good and surgery involves an attempt at hearing preservation in less than or equal to 10 mm VS, neuromonitoring of the VIII nerve is highly recommended. The best-known intraoperative monitoring modalities are brainstem auditory evoked response (BAER) and cochlear nerve action potential (CNAP) [29]; both use a cochlear electrode, but they differ in the positioning of the recording electrodes: while the first one uses electrodes located in the scalp, the latter uses electrodes located at the nerve entry zone of the brainstem.

CNAP testing provides real-time feedback with larger response potentials, representing a more reliable test than BAER [29]. When CI is an option, planned with hearing sacrifice or in failures of HPS (hearing preservation surgery) cases, the intraoperative testing of cochlear nerve function in deaf patients with an electric stimulus is still a work-in-progress method, the reliability of which is still to be defined. The anatomical integrity of the cochlear nerve is judged at the end of surgery as a prerequisite to cochlear implantation.

Another important aspect of CI is cochlear patency, mainly affected by cochlear manipulation during translabyrinthine surgery and delayed CI. According to Delgado et al., about 40% of MRI scans show some degree of cochlear ossification within the first year after translabyrinthine surgery, with a probable time-dependent process [30].

This condition can lead to potential electrode damage or even the abortion of the positioning of the electrode with the failure of CI. Wick et al. describe 8% intraoperative complications, specifically difficult CI insertions due to cochlear fibrosis in patients who underwent delayed cochlear implantation. Nearly 70% of the “delayed CI” group underwent HPS with the failure of hearing preservation (57% retrosigmoid approach; 11% middle fossa approach) with a median time delay of 8 months. Nevertheless, their recent meta-analysis showed that the timing of CI (delayed versus simultaneous) does not significantly affect auditory performance in terms of the ability to use the CI and the achievement of open-set speech.

These data show that a simultaneous CI operation should be performed when feasible, and in the case of a delay (i.e., HPS with the failure of hearing preservation), CI should be performed as soon as possible [20].

Arnoldner et al. proposed a scoring system to identify patients with greater changes in nerve integrity. Overall, the results indicated that patients with Class I-II (i.e., score >6) have good chances of positive intraoperative eABR and hence can be implanted (Table 2) [26].

### 3.2. Sporadic VS and CI Rehabilitation

Sporadic VS occurs mostly unilaterally with a common symptom of hearing loss that may be sudden or may progress slowly over time. The prevalence of VS in patients with sudden sensorineural hearing loss (SSNHL) who undergo MRI is 3.0%, which is 150 times greater than the prevalence of incidental VS [31]. Commonly, hearing loss from VS is attributed to the mechanical compression of the adjacent cochlear nerve, as shown by the presence of retro-cochlear abnormalities in ABR testing and cochlear nerve atrophy. Several histopathologic studies exhibit significant inner and outer hair cell loss, cochlear neuronal loss, and precipitates in the inner fluid, indicating that hearing loss in patients with VS may also be caused by cochlear degeneration [32]. A retrospective analysis of the temporal bones of patients with VS was conducted to highlight the decrease in the density of auditory-nerve fibers within the osseous spiral lamina (OLS) of different regions of the cochlea. A lower density in the OLS is observed when VS arises from the inferior vestibular nerve, and specifically, a greater loss in the upper basal turn corresponds to isolated hearing impairment for the frequency of 2 KHz. Interestingly, the contralateral spiral ganglion cell count in VS patients is below the average based on age with a risk of progression to moderate contralateral hearing loss [32]. Unilateral profound hearing impairment causes the loss of three functional advantages of binaural hearing: the head shadow effect, binaural summation, and binaural squelch. In addition to the improvement in speech perception gained from binaural hearing, sound localization also requires binaural hearing and is severely impaired in patients with single-sided deafness [15]. A recent prospective multicenter study showed that CI for single-sided deafness (SSD) and asymmetric hearing loss (AHL), which were previously treated with the contralateral routing of a signal hearing aid (CROS-HA) or a bone conduction device (BCD), specifically a bone-anchored hearing aid (BAHA), could provide better speech perception in spatially separated noise, better sound localization, a better quality of life (QoL), and a decrease in the severity and incidence of tinnitus [33].

The hearing results (pre- and postoperative PTA, SDS in quiet and with background noise) showed a significant increase in postoperative PTA and SDS in all groups and specifically proved the better performance of bimodal hearing stimulation compared to only CI use [34]. In these settings, several studies demonstrated that CI after VS resection could provide better speech perception in spatially separated noise, better sound localization, a better quality of life (QoL), and a decrease in the severity and incidence of tinnitus.

The first prospective study that explored the audiological outcomes of CI after translabyrinthine resection of sporadic VS was conducted by Rooth et al. Seven patients met the inclusion criteria (small tumors, surgery feasibility, and willingness to undergo postoperative rehabilitation) and underwent CI simultaneously; the main determinant for cochlear implantation was the surgeon’s perception of an intact cochlear nerve after schwannoma resection [16]. Five patients had auditory perception at the time of activation, while two remained without auditory perception. All of them demonstrated improvements in sound localization, speech perception in noise, tinnitus severity, and SSQ scores in speech and spatial domains. Interestingly, the study also shows that the improvements reached are less than in patients who undergo CI for idiopathic unilateral hearing loss, as there may be a period of sensory neuropraxia as the cochlear nerve recovers after tumor resection [16].

Sanna et al. conducted a prospective study with the largest series of patients and analyzed the hearing results and subjective benefits of cochlear implantation concurrent to tumor resection in sporadic VS when contralateral hearing is normal [15]. A total of 13 subjects underwent VS resection with a modified translabyrinthine approach with the preservation of the VIII cranial nerve and simultaneous cochlear implantation. The audiological tests performed in the follow-up were pure-tone audiograms and closed-set (vowel identification, VI) and open-set formats in free-field conditions with contralateral hearing masked with white noise, along with other tests evaluating binaural benefits, sound localization, and overall subjective improvements. A discrete improvement in the mean PTA was noted in low frequencies, which was not statistically significant (*p* > 0.05). Likewise, PTA, disyllabic word recognition, sentence recognition, and common phrase comprehension improved in the second control, but this improvement was not statistically significant (*p* > 0.05). Ninety percent of the patients reported using the CI between 5 and 7 days per week. The median satisfaction score on a 10-point scale was 8. The limitations of this study were the small, albeit the only existing, series of patients, short follow-up (13 months), and lack of intraoperative tests to assess cochlear nerve functionality [15].

Conway et al. showed similar outcomes in their most recent prospective study in terms of improved hearing performance in background noise, better spatial discrimination, and a decrease in tinnitus [14]. Given the level of evidence originating from prospective studies, the small number of patients enrolled in these studies supply outcomes that need to be critically analyzed. Several systematic reviews have been released in the last several years about CI outcomes after sporadic VS resection. The data from these studies are highly heterogeneous given the high number of case reports included and the lack of standardized tools to evaluate hearing performance after the implantation.

Tadokoro et al. reviewed 16 articles from 1995 to 2017, including 45 patients. The postoperative auditory outcomes were SDS 56.4 +/− 27.6% (vs. pre-op SDS 30.0 +/− 40.0%), PTA 28.8 +/− 8.3 dB (vs. pre-op PTA 79.8 +/− 32.7 dB), and AzBio 75.0 +/− 14.3%. A higher preoperative ipsilateral SDS speech discrimination score predicted a lower postoperative SDS, while tumor resection status, tumor location, the duration of deafness, ipsilateral PTA, and the timing of CI placement had a significant effect on patient outcomes. Additionally, among seven patients complaining of tinnitus, five noticed an improvement in symptoms [21].

In Thompson et al.’s systematic review on concurrent CI during VS exeresis, approximately 85% of subjects had auditory perception with their CI. A total of 75% of subjects were high to intermediate performers in terms of audibility, while most of the low performers still noted the subjective benefit of their implants and improvement from their preoperative condition [18].

Noteworthy are the preliminary results about IC in small non-growing VS without surgical resection. Seven patients with VS volumes from 0.11 to 1.02 cm^3^ were implanted after MRI surveillance for 2 months up to 7 years. All patients improved monosyllabic word recognition (from 6% to 55%) with a stable performance at the 12-month follow-up [24]. To summarize, the level of evidence for the presently included studies is low. Only a few studies are prospective, and none has a comparative setup, meaning that no studies have performed comparisons between single-stage and sequential procedures. Additionally, the high variability in the follow-up timing, audiological battery tests, and preoperative measurements accounts for implausible future meta-analyses and consequently necessitates a stronger level of evidence [27].

### 3.3. Neurofibromatosis Type 2 and CI

Four to six of all diagnoses of VS are associated with neurofibromatosis type 2 (NF2), an autosomal dominant condition caused by pathogenic variants in the NF2 gene on chromosome 22q [4]. This condition causes a greater risk of developing nervous system tumors, including VSs, meningiomas, and ependymomas, which can affect the brain and spinal cord, and peripheral nerve schwannomas. The diagnostic criteria for NF2 are bilateral VS or unilateral VS associated with one or more first-degree relatives with NF2. Since the evidence regarding this group derives from retrospective studies of small groups of subjects, the outcomes need to be critically considered. These patients benefit the most from the total surgical removal of VS when technically feasible; when not possible, partial resection with adjuvant radiotherapy can be taken into consideration [4]. Additionally, Bevacizumab treatment may be an option in terms of tumor control and hearing improvement for NF2 patients with progressive vestibular schwannoma considered poor candidates for surgery [35].

The resulting bilateral hearing loss, the tendency toward larger VS, and the higher recurrence rate represent a challenge in terms of hearing rehabilitation. Traditionally, the only option for patients with NF2 and severe to profound sensorineural hearing loss has been an auditory brainstem implant (ABI) [19].

Borsetto et al. evaluated hearing rehabilitation outcomes in CI recipients whose VSs were either observed or treated with radiotherapy. CI for hearing restoration in patients with stable VS is possible, regardless of whether tumor stability has been reached because of radiosurgery or because the tumor has stopped growing. Thus, radiotherapy seems not to influence CI outcomes, even though the follow-up in the majority of the studies was 12 months or shorter [23].

West et al. investigated the results of simultaneous tumor removal and cochlear implantation and compared NF2 to sporadic VS. Mean extrameatal tumors (0–40 mm) were not significantly different between the groups, but almost all NF2 tumors were in the CPA. Speech detection scores (SDS) were 65% for monosyllables, 58% for bisyllables, and 57% for sentences. The higher performers were those patients with preoperative hearing class A or B. There were no significant differences in SDS outcomes between NF2-associated and sporadic VS. Some tinnitus improvement and vertigo reduction were reported. Additionally, no association was found between the use of cochlear nerve testing and postoperative results, possibly due to the existence of time windows of 6–8 weeks for the cochlear nerve to recover after surgery manipulation [19]. Similar results in terms of PTA, SDS in quiet and in noise, and tinnitus improvement have been highlighted by Tian et al. Furthermore, it is not possible to make robust conclusions concerning the effects of the type, field, and dose of radiation on outcomes after CI (stereotactic radiosurgery, fractionated stereotactic radiosurgery, gamma knife surgery) [22].

In conclusion, CI seems to be effective even without the removal of the tumor, with reasonable hearing outcomes. The main requirement is a documented non-growing tumor. There is little consensus on the length of observation for untreated tumors before CI. A recent review encompassed these variables: the observation time extended from 1 to 3 years [25]. Reporting on the basis of the Danish national VS database, Stangerup et al. have shown that after 5 years of no tumor growth, further growth becomes unlikely [5].

Sorrentino et al. analyzed and correlated CI outcomes after VS surgery within a 24-month follow-up. Favorable CI outcomes at the 24-month follow-up with a high rate of daily users (77.8%) were in line with the literature. The NF2 group showed progressive improvement within 6–24 months after activation, underlining the importance of consistent audiological and speech rehabilitation over time. No correlation was found between good preoperative hearing and postimplant performance, while good preoperative contralateral hearing was revealed to be a negative prognostic factor for CI outcomes. In addition, patients with good contralateral hearing improved more gradually than patients with impaired contralateral hearing. This aspect highlighted the importance of preoperative patient selection, appropriate audiological counseling, and a well-structured rehabilitation plan, which should be tailored to patients’ needs and conditions. Therefore, similarly to what was proposed by other authors, CI after VS surgery is preferred when the preoperative hearing class is C worse. The option of HPS is preferred in good-hearing patients (A–B classes), setting aside CI when this attempt fails [17].

### 3.4. MRI Surveillance and CI

CI may affect the imaging surveillance of tumor progression or other disease-specific conditions. MRI is the gold standard for the imaging of VS. The main issues in performing MRI are distortional artifacts, safety, magnet displacement, and pain. Walton et al. [36] reported no adverse effects on the implant and an 85% unaltered view of the internal acoustic canal and cerebellopontine angle for patients who underwent 1.5 MRI using a protocol that includes infiltration with a local anesthetic and a head wrap with a plastic plate. Nowadays, most brands producing CI provide recommend MRI for patients. Specifically, the latest models of cochlear implants (COCHLEAR NUCLEUS PROFILE PLUS SERIES, MEDEL SYNCHRONY 2, and AB HIRES ULTRA 3D) are compatible with 3T MRI without magnet removal. Yet, distortional artifacts in MRI images may lead to a difficult assessment of the internal acoustic canal (IAC), cochlea, and cerebellopontine angle (CPA). According to Schwartz et al., the ipsilateral IAC was found to be visualized better in T1 with gadolinium sequences, with fewer artifacts than those noted in CISS images. This difference was not as pronounced when visualizing the CPA [37]. Sudhoff et al. reviewed postoperative 3T MRI images of patients who underwent VS resection with simultaneous CI (MedEL, Cochlear, Advanced Bionics), specifically positioned 7–9 cm behind the external auditory canal, with the cochlea and the IAC visually accessible [38]. A study analyzed the visibility of the inner ear structures in relation to the location of the magnet. The CI’s magnet was placed in nine different positions defined by the nasion–outer ear canal angle. The used angles were 90, 120, and 160 degrees. The distance of the magnet from the outer ear canal was 5, 7, and 9 cm. The best visualization of the IAC and the labyrinth was achieved mainly in two positions: at 90 degrees/9 cm and at 160 degrees/9 cm, projecting the center of the shadow area, respectively, behind and superior to the IAC [39]. Additionally, head positions during MRI acquisition may influence the distance of the artifacts: especially in the coronal plane, the antero-flexion chin-to-chest position of the head can improve the evaluation of the cochlea and the IAC [38].

### 3.5. Intralabyrinthine Schwannoma and CI

Intralabyrinthine schwannomas (ILSs) are very rare, with fewer than one thousand cases described in the literature. They usually arise in the modiolus and then seem to spread through three different patterns: the invasion of the cochlear basal turn, the erosion of the cribriform area, or intravestibular diffusion [40]. ILSs are an underestimated cause of vertigo and hearing loss; MRI with gadolinium is crucial for their diagnosis, since it can detect even very small tumors of 2–3 mm and help in the differential diagnosis of cochleovestibular disorders. Although positive outcomes have been demonstrated for cochlear implantation at the time of VS resection, some authors argue that there is a risk of cochlear nerve damage if a CI is placed without tumor resection. As ILSs demonstrate a low growth rate, as in other schwannomas, the “wait and scan” strategy is a safe approach, and the main issue is represented by hearing restoration [41].

For purely intracochlear schwannomas, Carlson et al. describe leaving the intracochlear tumor in situ to preserve the cochlear anatomy and physiological function while using a styleted electrode with the late deployment of the off-stylet electrode to aid in its advancement. A total of 80% of the affected ears achieved good open-set word recognition [42].

Laborai et al. report a small case series of patients with ILSs undergoing CI with a standard round window approach without tumor removal, showing it is a feasible option for binaural hearing restoration in these patients. The issue is debated regarding the long-term outcome with the possible growth of the residual VS and the issue of MRI monitoring of VS schwannoma growth [27].

Plontke et al. reported the audiological outcomes of four patients who underwent subtotal cochleoectomy for IVS removal and concurrent CI with a new, perimodiolar malleable electrode array; this surgical technique allows a more radical and safe removal of the tumor compared to other techniques (“double cochleostomy” with “pull-through” or “push-through” of the tumor), with a minor risk of residual tumor growth. Additionally, the pre-shaped malleable array allows the maximum approximation of electrode contacts with the spiral ganglion cells in Rosenthal’s canal with probable better long-term audiological outcomes [43].

Jia et al. retrospectively evaluated the audiological outcomes in a small case series of NF2 patients with ILSs. This study demonstrates that the removal of IVS with concomitant CI implantation provides a long-term benefit from CI, although it could be altered by the occurrence of other intracanalicular and/or CPA NF2-related tumors, the treatment of which might restore the initial CI benefit [28].

In Zanoletti et al. 2019, the transmeatal approach to remove small intrameatal VS with intralabyrinthine–intracochlear extension preferably excluded the possibility of hearing rehabilitation with CI, with the potential exception of intravestibular VS or small VS of the basal turn of the cochlea, where it seemed possible to remove the tumor and apply CI [44].

## 4. Conclusions

Cochlear implantation is an effective hearing rehabilitation option when good patient selection is conducted, and realistic expectations are foreseen. From many of the studies mentioned above, the best preoperative condition is indicative of the best postoperative results. Radiosurgery is effective in tumor control for selected patients, but the potential delayed effect of radiation on long-term hearing outcomes is unknown, mostly due to cochlear damage. Large VSs often need to be surgically removed with worse facial and cochlear nerve outcomes, and consequently, hearing rehabilitation with CI is not feasible since the cochlear nerve is rarely preserved. Early surgery seems to be preferable to delayed surgery when hearing preservation/rehabilitation is the goal. Natural hearing preservation with HPS is a preferable option to CI rehabilitation, but when the best conditions are not preoperatively maintained, CI rehabilitation is a promising option. Sequential CI may also be a choice in HPS failures. Our in-home recommendations that reflect these statements are shown in Table 3. In particular, CI after HPS failure should be considered as soon as possible (1–3 months) whenever major complications (early and late cerebellar edema, nasal or wound cerebrospinal fluid leak, wound hematoma) and RMI-assessed cochlear patency have been evaluated.

CI after translabyrinthine resection should be considered in small growing tumors when the cochlear nerve has been macroscopically conserved, even in the case of a negative intraoperative response, possibly due to sensory neuropraxia.

CI in NF2 patients with bilateral hearing loss should be considered after either surgical VS resection or radiotherapy. In the likelihood of worsened hearing or tumor recurrence in the implanted ear, Bevacizumab could be an option.

Lastly, patients with symptomatic intracochlear schwannomas who undergo surgical removal of the tumor may benefit from simultaneous CI when no radicality doubts exist, while sequential CI could be preferred after the placement of a dummy electrode to mitigate the risk of fibrosis.

To date, most of the existing studies are based on growing VSs that have already impaired cochlear nerve integrity or function. In our opinion, a paradigm shift should be undertaken considering the natural history and the long-term outcome of surgery; when a small VS is affected by a medium-severe degree of hearing loss, it should be treated earlier in order to obtain the best preconditions for simultaneous CI.

## Figures and Tables

**Table 1:** Summary of paper’s results.

Study	Study Design	Groups	Inclusion Criteria	Tumor Size (Mean)	Audiological Results	Follow-Up (Months)	Surgical Timing	Intraoperative Monitoring
Conway et al., 2021 [14]	PS	14 (sporadic VS)	Size < or equal to 2 cm	7 mm (3–16 mm)	Improvement in AzBIO quiet, +10, +5 SNR, and CNC affected	3	S (1 D)	no
Sanna et al., 2016 [15]	PS	13 (sporadic VS)	Hearing classes A and B; intrameatal tumor or Kanzaki class I, 18 > age	-	Discrete improvement in PTA (low frequencies), disyllabic word recognition, sentence recognition, and common phrase comprehension (*p* > 0.05); Improvement in binaural hearing and subjective questionnaires	6	S	no
Rooth et al., 2017 [16]	PS	7 (sporadic VS)	T size < 15 mm; contralateral PTA < or equal to 35 dB HL;	4–15 mm	Five out of seven had auditory perception; improvement in localization abilities; Improvement in AzBIO scores at 0 dB SNR; CNC did not demonstrate any improvement; Improvements in tinnitus (THI) and SSQ	6	S	no
Sorrentino et al., 2021 [17]	RS	17 (8 sporadic and 9 NF2-associated VSs)	Institution protocol for VS surgery and CI implantation	10 mm (9.3 in sporadic VS, 13.0 in NF2)	77.8% were active users; Median postoperative PTA 45.6 dB nHL, median WRS = 40%; median postoperative PTA in the implanted ear was better in the group with impaired contralateral hearing; Good preoperative contralateral hearing status was negative prognostic factor for CI performance	24	10 S, 7 Dto failed HPS	no
Thompson et al., 2019 [18]	RS	41 (29 sporadic VS, 12 NF2)	-	13 mm (2–40 mm)	85% of patients achieved audibility with CI (75% high-intermediate performers, 25% low performers); Improved sound localization and subjective benefits	-	-	-
West et al., 2019 [19]	RS	86 (53 sporadic VS, 33 NF2)	Simultaneous (single-stage) VS removal and ipsilateral cochlear implantation, original study types including case reports	18 mm (NF2) 5 mm (sporadic)	SDS mean performance scores (including patients with no audibility) were 65% for monosyllables, 58% for bisyllables, and 57% for sentences. Tumor size: 7 mm in high performers, 10 mm in low performers; 78% of the patients with preoperative hearing class A or B had postoperative high performance, while this was only the case for 51% of the patients with preoperative hearing class C or D; Improvement in tinnitus	-	-	-
Wick et al., 2020 [20]	RS	93 (46 in Group 1, simultaneous; 47 in Group 2, sequential)	-	Group 1: 12 mm; Group 2: 15 mm	GROUP 1 VS. GROUP 2 Open-set speech reached in 50% VS. 59.6%; Word score mean 52% VS. 37.9%; Sentence score mean 65.4% VS. 49.6%	32.3 (1); 36.9 (2)	46 S/47 D	3 cases eABR
Bartindale et al., 2021 [21]	RS	45 (sporadic VS)	(1) Sporadic VS, (2) CI placed on the side of the VS, (3) at least 6-month follow-up after their CI was placed, (4) CI auditory outcomes reported, and (5) patients with NF2 were excluded	-	Average SDS was 56.4% with a standard deviation of 27.6; AzBio testing (Arizona Biomedical Institute sentence test) showed an average of 75%. The average postimplant audiometry threshold was 28.8 dB; Neither tumor resection status, tumor location, duration of deafness, ipsilateral PTA, nor timing of CI placement had a significant effect on patient outcomes	Mean 20.2	S/D	ePS
Tian et al., 2021 [22]	RS	33	Ipsilateral cochlear implantation outcomes after radiotherapy (all types) of VS (both sporadic and NF2) were included.	20.5 mm	HINT mean score 71%; BKB mean score 52%; CNC test mean score 51%; CUNY mean score 78%; Postoperative PTA mean value 35 dB	Mean 18.5	-	ePS; eCAP
Borsetto et al., 2019 [23]	RS	50 (5 sporadic vs, 45 NF2) GROUP 1: RT Group 2: Observation	(1) Had a VS, either sporadic or NF2-associated, (2) had undergone CI, (3) had not undergone surgical treatment for their VS prior to CI, and (4) had hearing outcome measures post-CI reported. Patients who received Bevacizumab to control tumor growth after CI were included in this review.	21 mm (1)	The mean post-CI pure-tone average for the radiotherapy group was 33 ± 20 dB (*n* = 9) versus 39 ± 9 dB (*n* = 4) for the observation group. Furthermore, comparison of speech perception scores also revealed no consistent differences between the two groups (SRS, WRS, HINT, CUNY, CNC)	12 (most studies)	-	-
Longino et al., 2021 [24]	RS	7	VS managed with observation alone and underwent ipsilateral CI without prior surgery or radiotherapy	-	Average improvement in CNC word score preimplantation to 12 months postimplantation was 49% (range, 24–88%). Average improvement in AzBio of 45% when compared with preimplantation testing (range, 23–80%; *n* = 6).	12	-	-
Urban et al., 2020 [25]	RS	7 (4 sporadic VS, 3 NF2)	All cases of cochlear implantation in patients with unresected vestibular schwannomas	-	Mean postoperative sentence score was 63.9% (range 48–91). Average postimplant CNC word scores were available for five patients and averaged 59%.	28		
Arnoldner et al., 2021 [26]	Clinical Experience	10	Sporadic VS showing growth on repeat imaging studies, no ipsilateral functional hearing, desire to undergo cochlear implantation, and general good health.	-	Mean aided pure-tone average was 38 dB HL. Mean WRS was 28% at 65 dB and 52% at 80 dB.	6	S	eABR
Laborai et al., 2021 [27]	Case report	2 patients	Intralabyrinthine schwannoma without tumor removal	-	(1) PTA 35 dB; 21% word recognition at 65 dB, resolution of tinnitus, DHI decrease (2) PTA 30 db; 27% word recognition at 65 dB	6	-	-
Jia et al., 2019 [28]	Retrospective case review	3 patients	Patients with IVS in whom cochlear implantation had been performed.	-	(1) WRS: 90%; SRS: 100%, 87%, 73% in quiet, noiseþ10 dB SNR, and noiseþ5 dB SNR, respectively (2) WRS 76% and SRS 100%, 80%, 80%, 33% in quiet and in noise, þ10, þ5, and 0 dB SNR, respectively (3) 90% WRS and 100% SRS at 1 year versus 60% WRS and SRS at 3 years	60 (except 3 patient)	S	-

PS: Prospective Study; RS: Retrospective study; S: Simultaneous; D: Delayed; PTA: pure-tone average; CNC: consonant-nucleus-consonant; THI: Tinnitus Handicap Inventory; SSQ: Speech, Spatial and Qualities of Hearing Scale; AzBio: Arizona Biomedical Institute sentence test; SDS: speech discrimination score; ECAP: electrically evoked compound action potential; EPS, electrical promontory stimulation; eABR: electrical auditory brainstem response; BKB, Bamford–Kowal–Bench; CUNY, City University of New York sentences; HINT, Hearing in Noise Test; IVS: intravestibular schwannoma.

**Table 2:** Arnoldner et al. score [26].

Category	Definition	Points
Koos Grading	Koos 4 Koos 3 Koos 2 Koos 1	0 1 2 3
Extension	Transmodiolar extension Infiltration of modiolus Contact with modiolus No contact, no infiltration of modiolus	0 1 2 3
Hearing	Complete hearing loss Some residual hearing (0% monosyllables) ≥1% monosyllables, any PTA	0 1
PS EABR	No response Unclear wave V Stable wave V	0 1 2
Total score	Class IV Class III Class II Class I	0–3 4–5 6–7 8–10

Scoring system to identify patients with higher chances of nerve integrity in case of VS resection. A certain amount (0–3) of points are given in four categories. Points are added up and patients are categorised to a certain class which reflects the probability of cochlear implantation after translabyrinthine vestibular schwannoma excision. Promontory stimulation eABR (PS EABR).

**Table 3:** In-home recommendations.

Tumor Size (mm)	Decision Factors: Hearing (Tokyo Hearing Class) and/or Growth	Treatment
T < 10	Good hearing [< 30 PTA/>70% SDS (A-B Tokyo)]	Hearing preservation surgery (or Observation) (CI in HPS failures)
	Good hearing surgical risk, not growing tumors; Good hearing surgical risk, growing tumors	Observation; Radiotherapy
	Bad hearing (C-D Tokyo), not growing tumors. Bad hearing, growing tumors	Observation; Surgery and CI rehabilitation
T 10–15	Not growing tumors	Surgery (or Observation)
	Growing tumors	Surgery
	Not growing tumors + surgical risk Growing tumors + surgical risk	Observation; Radiotherapy
T 15–25	-	Surgery or Radiotherapy
T > 25	-	Surgery
	Surgical risk	Partial Surgery and Radiotherapy
Any Size	Cystic tumor	Surgery

## Data Availability

The data presented in this study are available on request from the corresponding author.
